# Identification and pathogenicity of *Fusarium* spp. associated with tea wilt in Zhejiang Province, China

**DOI:** 10.1186/s12866-023-03174-4

**Published:** 2024-01-27

**Authors:** Zhaoyang Tang, Jing Zhu, Qiujin Song, Paul Daly, Liya Kong, Luqian He, Agen Li, Jun Lou, Zhanqi Wang, Liqin Zhang, Lijing Min

**Affiliations:** 1 Key Laboratory of Vector Biology and Pathogen Control of Zhejiang Province, College of Life Sciences, Huzhou University, Huzhou, 313000 China; 2 Zhejiang Zhongyi Testing Institute Co., Ltd., Huzhou, 313000 China; 3 Institute of Plant Protection, Jiangsu Academy of Agricultural Sciences, Nanjing, 210014 China; 4 Yuhang Agro-Ecological Environment & Crop Protection Service Station, Hangzhou, 310000 China

**Keywords:** Tea wilt, *Fusarium*, Pathogenicity, *Fusarium fujikuroi*

## Abstract

**Background:**

Tea is one of the most widely consumed beverages in the world, with significant economic and cultural value. However, tea production faces many challenges due to various biotic and abiotic stresses, among which fungal diseases are particularly devastating.

**Results:**

To understand the identity and pathogenicity of isolates recovered from tea plants with symptoms of wilt, phylogenetic analyses and pathogenicity assays were conducted. Isolates were characterized to the species level by sequencing the ITS, *tef-1α*, *tub2* and *rpb2* sequences and morphology. Four *Fusarium* species were identified: *Fusarium fujikuroi*, *Fusarium solani*, *Fusarium oxysporum*, and *Fusarium concentricum*. The pathogenicity of the *Fusarium* isolates was evaluated on 1-year-old tea plants, whereby *F. fujikuroi* OS3 and OS4 strains were found to be the most virulent on tea.

**Conclusions:**

To the best of our knowledge, this is the first report of tea rot caused by *F. fujikuroi* in the world. This provides the foundation for the identification and control of wilt disease in tea plants.

## Introduction

Tea (*Camellia sinensis* [L.] O. Kuntze) is a prominent plantation crop that belongs to the family Theaceae [1], which is cultivated in subtropical regions across Asia, Africa, and South America. As one of the most widely consumed beverages globally, tea holds significant importance in the global market [2]. In 2022, China was the world’s largest producer of tea, producing 2.8 million tonnes [3], with the majority of tea produced by smallholder farmers, who significantly benefited financially from the tea industry.

Despite its prominence, tea cultivation faces various biotic threats, and fungal diseases are the most serious, as they can cause significant losses in yield and quality. Among these diseases, blister blight, gray blight, brown blight, twig dieback, stem cankers, and root rots are considered to be the most severe [4]. In particular, the genus *Fusarium* comprises soil-borne fungi known to cause wilt, leaf spot, collar canker, and dieback in tea plants [5, 6]. Fusarium collar canker and dieback are caused by *Fusarium solani*, which infects the stem and causes cracks, peeling of bark, white mold growth and eventual branch death in China and Sri Lanka [6, 7]. *Fusarium proliferatum* induces Fusarium leaf spot, characterized by the emergence of brown lesions with yellow halos on tea leaves [5]. Additionally, alongside *Fusarium*-caused tea root diseases, *Poria hypolateritia* acts as a fungal pathogen responsible for red root-rot disease, while *Phomopsis theae* causes Phomopsis (collar) stem canker disease, both posing significant concerns that impact the growth, yield, and quality of tea plants [8, 9]. These diseases can be severely detrimental to tea production, resulting in notable losses in both yield and quality for tea growers. Therefore, effective monitoring and control measures must be implemented to manage *Fusarium* diseases on tea plants.

During a survey of tea plantations in Zhejiang, we observed that some tea trees exhibited signs of wilt, and we collected diseased samples and isolated and identified the pathogens responsible for the wilt of the tea trees. This research focuses on the identification and pathogenicity associated with tea rot. By gaining a deeper understanding of the specific *Fusarium* species, the tea industry can implement more targeted and effective management strategies.

## Materials and methods

### Sample collection and fungal isolation

Tea plants with rot were collected from Anji, Huzhou and Yuhang, Hangzhou tea plantations. Fungal strains were isolated following the method described by Tang [10]. The diseased root and stem samples were surface disinfected with 75% ethanol for 1 min followed by 0.5% sodium hypochlorite solution for 2 min and then soaked in sterile distilled water three times to remove the sterilizing agent. Samples were dried by blotting, and disease tissues were cut into small pieces (3 mm^2^) and then placed on potato dextrose agar (PDA) (BD Difco) with ampicillin (50 µg/mL) and rifampicin (25 µg/mL) antibiotics. The plates were incubated at 25 °C until hyphae emerged from the tissue. A small amount of fresh hyphal tips were cut and transferred onto PDA and incubated at 25 °C.

### DNA extraction, PCR, and sequencing

The isolates were used for DNA extraction and sequencing. For each isolate, the mycelium taken from a 7-day-old culture grown on PDA was frozen and homogenized. Genomic DNA was extracted using the 2% cetyltrimethylammonium bromide (CTAB) method [11]. PCR amplification was performed for the internal transcribed spacer region (ITS) of ribosomal DNA, *translation elongation factor 1-alpha* (*tef-1α*), *beta-tubulin* (*tub2*), and *RNA polymerase II second largest subunit* (*rpb2*). The primer pairs used in this study are listed in Table 1. All PCR products were subjected to electrophoresis in 2% agarose gel, purified by a gel extraction kit (Vazyme, Nanjing, China), and sequenced by the dideoxy termination method at Sangon Biotech (Shanghai) Co., Ltd, China. The ITS, *tef-1α*, *tub2* and *rpb2* gene sequences were deposited in GenBank (Table 2).

**Table 1:** The primer pairs used in this study

Gene	primer pairs		References
internal transcribed spacer region (ITS) of ribosomal DNA	ITS1: CTTGGTCATTTAGAGGAAGTAA ITS4: TCCTCCGCTTATTGATATGC		[22]
translation elongation factor 1-alpha (*tef-1α*)	EF-1: ATGGGTAAGGARGACAAGAC EF-2: GGARGTACCAGTSATCATGTT		[23]
beta-tubulin (*tub2*)	Bt-F: AACATGCGTGAGATTGTAAGT Bt-R: TCTGGATGTTGTTGGGAATCC		[24]
RNA polymerase II second largest subunit (*rpb2*)	RPB2-5F2: GGGGWGAYCAGAAGAAGGC RPB2-11aR: GCRTGGATCTTRTCRTCSACC		[25, 26]

**Table 2:** Details of the *Fusarium* spp. species sequences used in the molecular phylogenetic analysis

Strain	ITS	*tef-1α*	*tub2*	*rpb2*
FS4	OR364054	OR387127	OR387135	-
FS5	OR364055	OR387128	OR387136	OR387143
bj2	OR364056	OR387129	OR387137	OR387144
FS7	OR364057	OR387130	OR387138	OR387145
OS3	OR364058	OR387131	OR387139	-
OS4	OR364059	OR387132	OR387140	-
aj2	OR364060	OR387133	-	OR387146
ej2	OR364061	OR387134	OR387141	-
*Fusarium fujikuroi* CBS 221.76		AB725605	AB725606	KU604255
*Fusarium proliferatum* CBS 480.96		MN534059	MN534129	MN534272
*Fusarium sacchari* CBS 223.76		MW402115	MW402313	JX171580
*Fusarium concentricum* CBS 450.97		AF160282	MW402334	JF741086
*Fusarium mangiferae* CBS 120,994		MN534017	MN534128	MN534271
*Fusarium fujikuroi* CBS 130,402		MW402025	MN534131	MN534269
*Fusarium fujikoroi* NRRL 13,289		MW402158	–	MW402777
*Fusarium fujikuroi* CBS 263.54		MW402121	MW402319	–
*Fusarium globosum* CBS 428.97		KF466417	MN534124	KF466406
*Fusarium annulatum* CBS 258.54		MT010994	MT011041	MT010983
*Fusarium acutatum* CBS 402.97		MW402125	MW402323	MW402768
*Fusarium nygamai* CBS 749.97		MW402151	MW402352	EF470114
*Fusarium pseudoanthophilum* CBS 414.97		MW402128	MW402326	–
*Fusarium fractiflexum* NRRL 28,852		AF160288	AF160315	LT575064
*Fusarium siculi* CBS 142,222		LT746214	LT746346	LT746327
*Fusarium secorum* NRRL 62,593		KJ189225	–	–
*Fusarium fredkrugeri* CBS 144,209		LT996097	LT996118	LT996147
*Fusarium dlaminii* CBS 119,860		MW401995	MW402195	KU171701
*Fusarium agapanthi* NRRL 54,463		KU900630	KU900635	KU900625
*Fusarium mexicanum* NRRL 53,147		GU737282	GU737494	MN724973
*Fusarium ophioides* CBS 118,512		MN534022	MN534118	MN534303
*Fusarium begoniae* CBS 452.97		MN533994	MN534101	MN534243
*Fusarium fracticaudum* CMW 25,245		KJ541059	KJ541051	PDNT00000000
*Fusarium ananatum* CBS 118,516		LT996091	MN534089	LT996137
*Fusarium anthophilum* CBS 222.76		MW402114	MW402312	MW402811
*Fusarium temperatum* MUCL 52,463		–	MW402359	MW402776
*Fusarium subglutinans* CBS 747.97		MW402150	MW402351	MW402773
*Fusarium awaxy* LGMF1930		MG839004	MG839013	MK766941
*Fusarium sterilihyphosum* NRRL 25,623		MN193869	AF160316	MN193897
*Fusarium guttiforme* CBS 409.97		MT010999	MT011048	MT010967
*Fusarium solani* CBS 101,018	LR583770	LR583651		LR583878
*Fusarium solani* GJS 09-1466	KT313633	KT313611		KT313623
*Fusarium cf. solani* CBS 124,893	JX435191	JX435141		JX435241
*Fusarium solani* NRRL 31,168	DQ094395	DQ246922		EU329563
*Trichoderma harzianum* CBS 226.95	AY605713	AY605833		AF545549

### Phylogenetic analyses

The DNA sequences of ITS, *tub2*, *tef-1α*, and *rpb2* were used in the phylogenetic analyses. Closest matches were identified by BLAST (Basic Local Alignment Search Tool) searches in the National Center for Biotechnology Information (NCBI) database (https://blast.ncbi.nlm.nih.gov/Blasnt.cgi) of sequences available in GenBank. Reference sequences of the *F. fujikuroi* species complex (FFSC), *Fusarium oxysporum* species complex (FOSC), and *Fusarium solani* species complex (FOSC) were downloaded from GenBank following references and are listed in Table 2. The MAFFT v. 6.864b (https://mafft.cbrc.jp/alignment/server/index.html) online tool was used to obtain multiple sequence alignments, which were visually inspected and improved manually when necessary. The tree was generated on MEGA 11 with default parameters and bootstrapping with 1000 replicates to obtain the maximum-likelihood (ML) tree [12].

### *Fusarium* morphological observations

For morphological characterization, mycelial discs (3 mm in diameter) were taken from the growing edge of 5-day-old cultures in triplicate, inoculated onto fresh plates of PDA and incubated in the dark at 25 °C. After a 5-day incubation period, the shape, color, and density of colonies were recorded. For the morphological characterization of fungal conidia, fungal strains were grown in 100 mL portions of potato dextrose broth (PDB) at 25 °C for 4 days, and the spores were collected by culture filtration using two layers of cheesecloth. Spores were centrifuged and resuspended in ddH_2_O at a concentration of 1 × 10^5 mL^− 1^. The shape, color, and size of the conidia were observed using light microscopy (Leica ICC50 W, Germany). The isolates were cultured on mung bean culture medium to observe macroconidia.

### Tea plant pathogenicity assay

The *Fusarium* isolates were tested for pathogenicity following the chaff-grain medium method described by Leslie and Summerell, with modifications [11]. Briefly, cereal chaff and grain were mixed together in an approximately 5:1 ratio, and then 200 mL tap water was added and mixed thoroughly to release any air bubbles and placed at 4 °C overnight. The chaff-grain mixture was wrapped in cheesecloth and drained until no more water could be released. The mixture was distributed into Erlenmeyer flasks and autoclaved for 15 min on two successive days. Containers were inoculated with conidial suspensions (10^5 cfu/mL) at a rate of 1 mL suspension per 100 mL chaff-grain mixture. The inoculated material was shaken vigorously and then incubated at 25 °C for 10 days until the medium was completely colonized. The culture was removed from the container and air-dried at room temperature overnight. Pathogenicity tests were conducted on one-year-old tea plants of the *Camellia sinensis* ‘Huangjinya’ cultivar. In pots, the inoculum was mixed with disinfected vermiculite at a rate of 2% of the final total volume before planting. The inoculated tea tree pots were placed under field conditions for tea plants, and each treatment was watered in a standardized manner. The status of the tea trees was then observed and recorded. The pathogen was reisolated from the tree after the development of symptoms.

## Results

### Disease sample symptoms

**Fig. 1:**
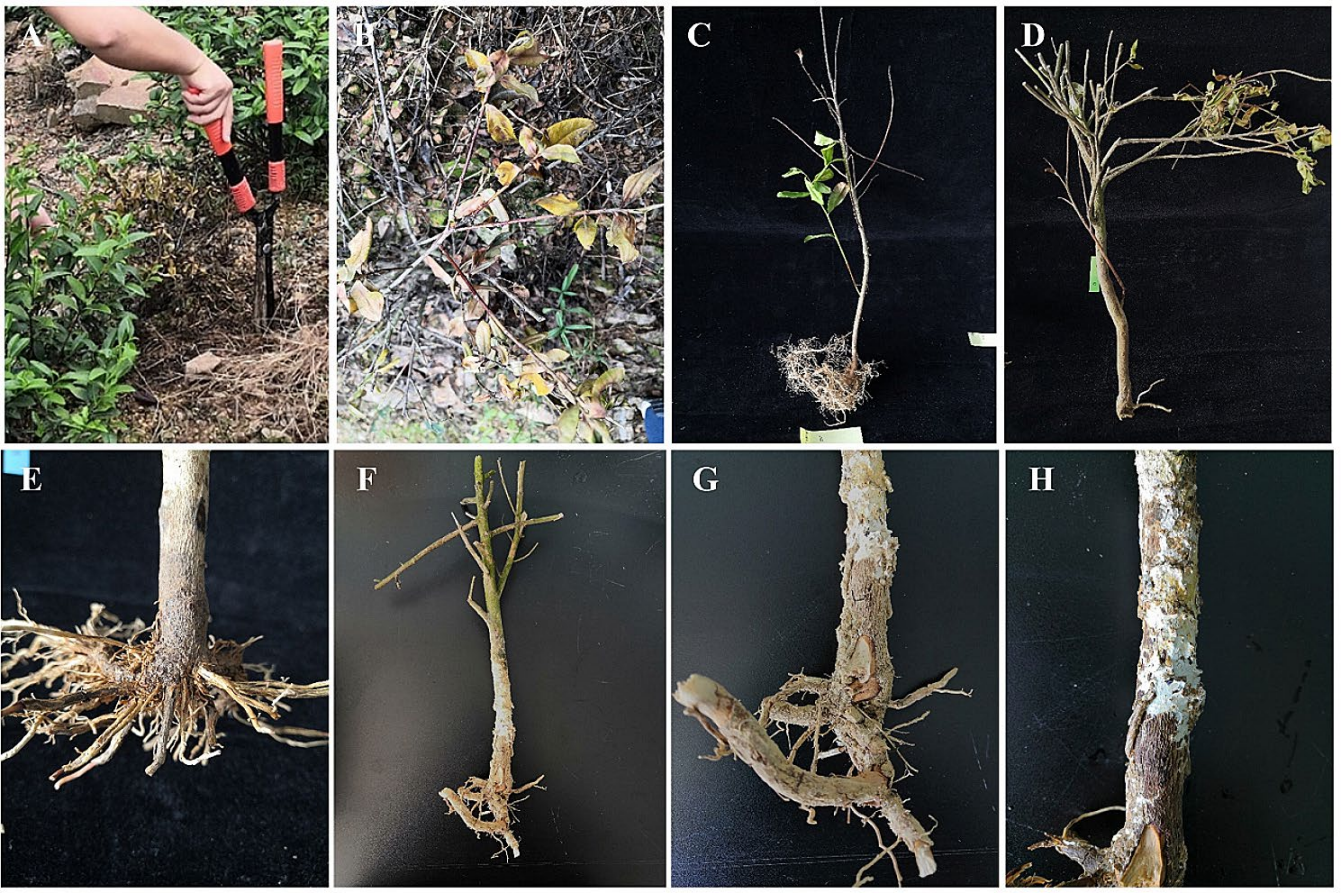
Symptoms of wilt diseases observed on tea trees in the plantation. (**A**-**B**) Wilt diseases caused death of the entire tea plant. (**C**-**D**) Wilt caused some branches to show wilt symptoms. (**E**) Root system browning and decay. (**F**) Infected plant of tea shown wilt. (**G**-**H**) Enlarged image of the roots and stems of the tea plant in **F**

During the autumn of 2020, tea bushes in the tea plantations of Anji, Huzhou (119.87970 E, 30.79132 N) and Yuhang, Hangzhou (119.4613 E, 30.2916 N), situated in Zhejiang Province, China, exhibited a range of diseased symptoms with 9% and 15% incidence rates from ‘Huangjinya’ cultivar. These symptoms were characterized by a reduction in leaf growth, yellowing of leaves, cankers exhibiting visible cracks, peeling of bark, progressive dieback of branches, and eventual death of the entire tree (Fig. 1). The cross-section of the infected stems showed that the vascular bundles turned gray and brown, while healthy plants had lighter colored vascular bundles. The severity of these symptoms suggests the presence of a potential pathogen, prompting the need for further investigation and intervention to prevent the spread of the disease.

### Isolation of causal agents

We isolated fungal strains from 10 tea plants showing symptoms of wilt and rot disease. A total of 73 fungal isolates were obtained from the roots and stems of diseased plants. These isolates were subjected to molecular identification using the ITS gene sequences by BLAST searches in the NCBI database. 15 isolates of *Fusarium*, 6 isolates of *Botryosphaeria*, and 52 isolates of *Trichoderma, Penicillium*, *Mortierella*, *Poitrasia*, and *Neurospora* were identified. Unexpectedly, for a tea plant that had been dead for a considerable period, we were able to isolate multiple strains of *Fusarium* from the woody tissues of the tea plants (Fig. 1F-H). To further identify these isolates at the species level, a random selection of eight isolates was subjected to additional analysis.

### Morphological observations

**Fig. 2:**
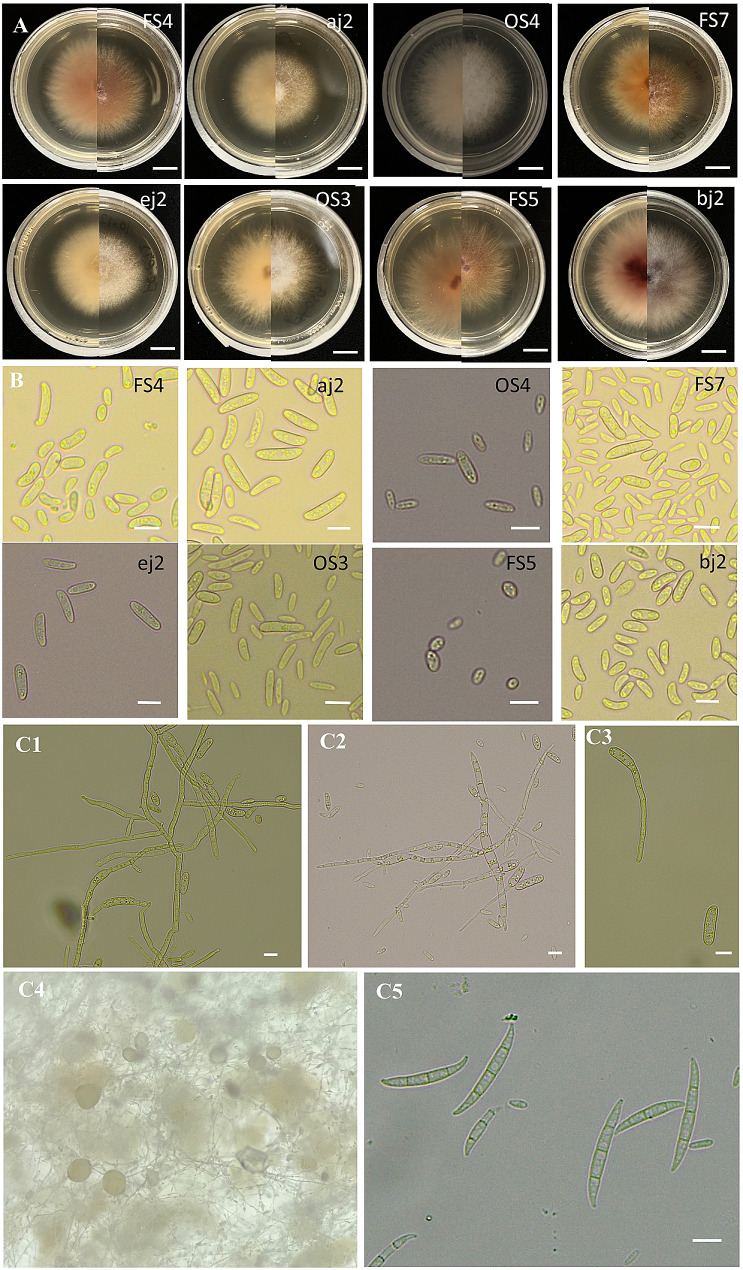
Morphological characters of *Fusarium* species. (**A**) Fungal colonies (reverse view on the left and surface view on the right) growing on PDA after 5 days of incubation period at 25℃. (**B**) Microconidia. (**C**) *Fusarium fujikuroi* OS3 isolate. (**C1**-**C2**) Conidiophores and conidiogenous cells. (**C3**) Microconidia and germinating microconidium. (**C4**) Sporodochia on mung bean culture medium. (**C5**) Macroconidia. (**A**) Bars = 1 cm; (**B**-**C**) Bars = 10 μm

Eight fungal isolates (FS4, FS5, FS7, aj2, bj2, ej2, OS3, and OS4) were selected and used in this experiment. Fungal colonies of each isolate were observed on PDA at 25 °C for 5 days. Colonies of FS4, FS5, FS7 and bj2 were cottony, elevated, azonate, white, turning pale salmon from the center with age with a white entire margin. Colonies of OS3 and OS4 were turning pale salmon from the center after 7 days. Aerial microconidia of the species FS4, FS5, FS7, aj2, bj2, ej2 were abundant, hyaline, kidney-shaped, aseptate. Microconidia of OS3 were hyaline, obovate, ellipsoidal to short falcate, smooth- and thin-walled, aseptate, 7.1 − 19.1 × 2.3–5.5 μm (av. 12.1 × 3.5 μm), clustering in false heads at the tip of phialides (Fig. 2). Macroconidia of OS3 were hyaline, falcate, with a foot-shaped basal cell, 3–4-septa, produced by prostrate phialides; 3-septate conidia 22.7–44.8 × 3.3–5.0 μm (av. 33.2 × 4.2 μm); 4-septate conidia 36.4–40.7 × 3.0–3.5 μm (av. 39.2 × 3.3 μm). Sporodochia of OS3 were densely aggregated, irregularly and verticillately branched, subhyaline, superficial or aerial (Fig. 2).

### Identification by DNA barcoding and phylogenetic analysis

**Fig. 3:**
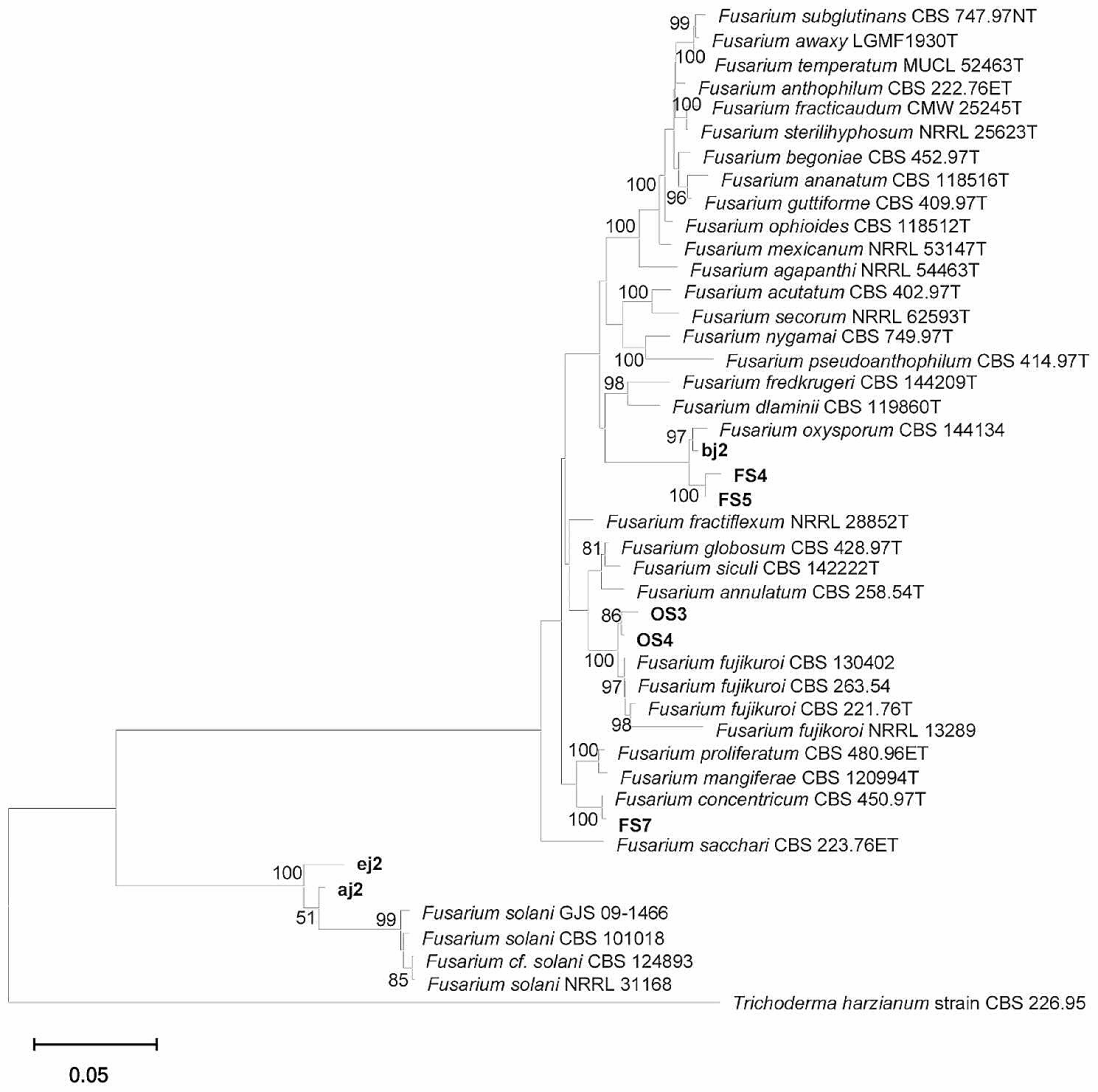
Evolutionary analysis by the maximum likelihood method. The percentage of trees in which the associated taxa clustered together is shown below the branches. The bootstrap consensus tree inferred from 1000 replicates is taken to represent the evolutionary history of the taxa analyzed. Evolutionary analyses were conducted in MEGA11. The tree of the *Fusarium* spp. analyses by ITS, *tef-1α*, *tub2*, and *rpb2* sequence data. The tree is rooted to *Trichoderma harzianum* strain CBS 226.95

*Fusarium* isolates were subjected to further molecular identification using *tub2*, *tef-1α*, and *rpb2* gene sequences. The isolates were identified as *F. oxysporum* (8 isolates), *F. fujikuroi* (2 isolates), *F. solani* (4 isolates) and *Fusarium concentricum* (1 isolate). The sequences derived from the eight fungal isolates obtained in this investigation were submitted to the GenBank database (Table 2). Based on the BLAST results and multigene phylogenetic analyses, strains OS3 and OS4 were identified as *F. fujikuroi*, and FS7 was identified as *Fusarium concentricum*, belonging to the *F. fujikuroi* species complex (FFSC). Fungal isolates FS5, bj2 and FS4 were identified as *F. oxysporum* belonging to the *F. oxysporum* species complex (FOSC). Additionally, strains aj2 and ej2 belonged to the *F. solani* species complex (FSSC). The phylogenetic tree was constructed in this study (Fig. 3), and the results of the phylogenetic analyses revealed topological patterns across the tree with strong bootstrap support for the identification of the isolates.

### Pathogenicity of some isolates confirmed by Koch’s postulates

**Fig. 4:**
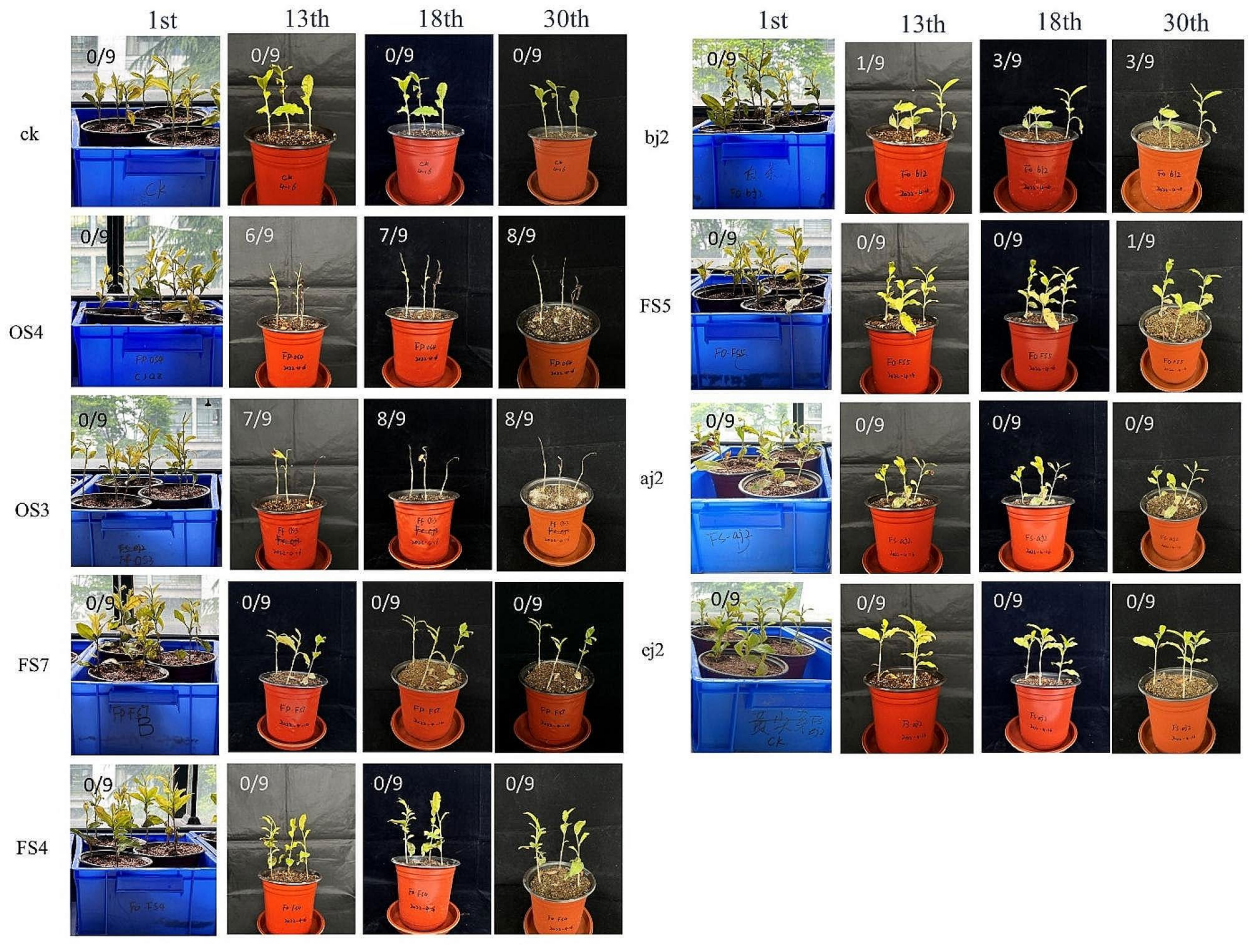
Pathogenicity assays. Symptoms of seedlings inoculated with *Fusarium* strains at 13, 18 and 30 days. CK indicates the control, and e.g., a score of 0/9 means 0 died and 9 plants in total were inoculated with the isolate

The disease symptoms on tea inoculated with the *Fusarium* isolates are shown in Fig. 4. Eight *Fusarium* isolates were tested for pathogenicity following the chaff-grain medium method. Each treatment group comprised three pots, with each pot housing three one-year-old tea seedlings, totaling nine seedlings per group. The experiments were conducted independently and repeated three times. Throughout the 30-day duration of each experiment, careful observations were made, and photographs were taken to document the condition of the tea plants. For the control group, the tea seedlings remained healthy throughout the entire period. For the *F. fujikuroi* OS3 and OS4 strains, the tea seedlings exhibited robust growth during the first six days. However, on the ninth day, some of the leaves began to turn brown, and the tender leaves showed signs of wilting. By the 13th day after inoculation, the OS3 (6 out of 9)- and OS4 (7 out of 9)-inoculated plants showed moderate infections, characterized by rot symptoms. After 30 days after inoculation, the plants displayed severe infections (8 out of 9). The *F. oxysporum* bj2 strain-inoculated plants displayed three deaths after 30 days of inoculation. The *F. oxysporum* FS5 strain only resulted in the mortality of one tea seedling. In contrast, no disease symptoms were observed on tea plants treated with FS7, FS4, aj2, and ej2 (Fig. 4). Each fungal isolate was consistently re-isolated from inoculated tissues and reidentified using sequencing methods of characterization to fulfill Koch’s postulates. The OS3 and OS4 strains are the same species belonging to *F. fujikuroi*, and both can cause tea wilt disease in tea plants. For the dead seedlings which were inoculated with either bj2 or FS5, the attempts to re-isolate the strains from these infected plants were not successful. To our knowledge, this is the first report of *F. fujikuroi* pathogenic fungus in tea plants.

## Discussion

This study was dedicated to identifying and comprehending the pathogenicity of fungal diseases responsible for tea rot. The results revealed that the *F. fujikuroi* isolates were the most aggressive in causing tea rot. We also isolated a number of other strains of *Botryosphaeria dothidea*, and strains from genera of *Penicillium*, *Mortierella* and *Trichoderma.* These isolates should be analyzed in future studies to test their pathogenicity to tea. Some *Trichoderma* were isolated from the fine and main roots of a dead tea tree. The isolated *Trichoderma* may have potential for biological control on the *F. fujikuroi* OS3 and OS4 disease. Previous research has extensively investigated the potential of *Trichoderma* in mitigating *Fusarium* wilt disease in tomato, soybean and banana [13–15].

The *F. fujikuroi* OS3 isolates were confirmed based on morphological characteristics and molecular identification. As shown in Fig. 2, the macroconidia of OS3 showed between 3 and 4 septa, and we did not find any macroconidia with five septa. The lack of observation of the macroconidia with five septa may be related to particular media components and cultures older than those in our study, and these older cultures may induce the macroconidia with five septa [16]. *F. fujikuroi* was previously reported to be associated with rice bakanae disease [17], soybean root rot [18], cotton seedling wilt [19], and root rot of tobacco [20]. This broad range of host associations underscores the adaptability and versatility of *F. fujikuroi* as a pathogen. Its presence in various agricultural systems highlights its capacity to exploit diverse plant hosts and potentially cause severe economic losses. Pine- and grass-associated species of the *F. fujikuroi* species complex were found to have two sets of genes which showed large differences in their ancestral origins, and they tended to occur in sub-telomeric regions of chromosomes underpinning the capacity of these fungi to colonize their respective plant hosts [21]. Future studies could test whether the tea infecting *F. fujikuroi* isolates from our study cause disease in grasses such as rice, and could use a comparative genomics approach to explore the molecular basis of the plant–fungus interactions. It is worth noting that this is the first report of tea rot caused by *F. fujikuroi* worldwide, indicating the importance of further research and monitoring for this emerging pathogen.

In conclusion, this study sheds light on the challenges posed by fungal diseases in tea production and highlights the importance of comprehensive pathogenicity assessments and identification of emerging pathogens. The discovery of *Fusarium* strains pathogenic to tea tree could lead to a promising avenue for managing tea plant wilt disease.

## Data Availability

All data and material are available upon request to correspondence author. All data has already been deposited in the National Center for Biotechnology Information (NCBI) database (www.ncbi.nlm.nih.gov/search/), and were assigned the accession numbers that list in Table 2.
